# Evaluation of Minimum Preparation Sampling Strategies for Sugarcane Quality Prediction by vis-NIR Spectroscopy

**DOI:** 10.3390/s21062195

**Published:** 2021-03-21

**Authors:** Lucas de Paula Corrêdo, Leonardo Felipe Maldaner, Helizani Couto Bazame, José Paulo Molin

**Affiliations:** Precision Agriculture Laboratory, Biosystems Engineering Department, ‘Luiz de Queiroz’ College of Agriculture, University of São Paulo, Av. Pádua Dias 11, 13418-900 Piracicaba, Brazil; leonardofm@usp.br (L.F.M.); helizanicouto@usp.br (H.C.B.); jpmolin@usp.br (J.P.M.)

**Keywords:** chemometrics, proximal sensing, precision agriculture

## Abstract

Proximal sensing for assessing sugarcane quality information during harvest can be affected by various factors, including the type of sample preparation. The objective of this study was to determine the best sugarcane sample type and analyze the spectral response for the prediction of quality parameters of sugarcane from visible and near-infrared (vis-NIR) spectroscopy. The sampling and spectral data acquisition were performed during the analysis of samples by conventional methods in a sugar mill laboratory. Samples of billets were collected and four modes of scanning and sample preparation were evaluated: outer-surface (‘skin’) (SS), cross-sectional scanning (CSS), defibrated cane (DF), and raw juice (RJ) to analyze the parameters soluble solids content (Brix), saccharose (Pol), fibre, pol of cane and total recoverable sugars (TRS). Predictive models based on Partial Least Square Regression (PLSR) were built with the vis-NIR spectral measurements. There was no significant difference (*p*-value > 0.05) between the accuracy SS and CSS samples compared to DF and RJ samples for all prediction models. However, DF samples presented the best predictive performance values for the main sugarcane quality parameters, and required only minimal sample preparation. The results contribute to advancing the development of on-board quality monitoring in sugarcane, indicating better sampling strategies.

## 1. Introduction

Near-Infrared (NIR) spectroscopy is a well-established technique to monitor the quality of raw sugarcane received by sugar mills [1], and consequently, for pricing and trading with producers and growers [2]. Crop quality is estimated based on physicochemical parameters related to physiological composition, such as soluble solids content (Brix), water-insoluble solids (Fibre), and the apparent sucrose in the juice (Pol). Furthermore, all other parameters (purity, Pol of cane, reducing sugars, and total recoverable sugars) are calculated based on the former parameters [3], from which total recoverable sugars (TRS) are used for the pricing and trading of the raw material. Sugarcane quality parameters are determined by analytical methods and empirical equations described by the National Council of Sugarcane Producers (CONSECANA), which are based on the International Commission for Uniform Methods of Sugar Analysis (ICUMSA). Using calibration methods, it is now possible to obtain some crop quality parameters by NIR spectroscopy [4].

Different wavelength regions of the electromagnetic spectrum can be used in spectroscopy, such as visible (400 to 750 nm), near-infrared (NIR, 750 to 2500 nm), shortwave near-infrared (SWNIR, 750 to 1100 nm), and visible and near-infrared (vis-NIR, 400 to 2500 nm) [5,6,7,8]. However, there is no consensus on the limits between these regions. Interaction between electromagnetic radiation and matter causes molecular vibrations involving heavy atom (C, N, O, and S) attached to a hydrogen atom [8]. This basic principle has allowed substantial scientific advances to predict organic compounds of agricultural products associated with its quality using vis-NIR spectroscopy as a nondestructive and environmentally friendly analysis technique [5]. Moreover, several studies have shown promising results when using the technique to predict sugar cane quality in the sugar mill [9,10,11,12] and for breeding programs [13,14,15].

Despite the advances in industrial sugarcane quality monitoring, spectral methods are still a distant reality for in-field measurements in line with precision agriculture practices. Current proximal sensing technologies applied at the canopy level allow only monitoring crop yield [16,17]. However, some studies have indicated that vis-NIR could also be a viable technology for acquiring quality data of harvested products in real-time during mechanical harvesting [16]. The monitoring of crop quality parameters across the field is important to adopting precision agriculture (PA) practices, in which quality maps would show the variability of the crop and help guide site-specific management [18]. In this context, mechanical harvest opens a way to obtain a high frequency of sampling and data collection to analyze the quality variability across the field [19]. However, some requirements need to be satisfied to use vis-NIR as proximal sensing technology for this purpose: (i) the location of adaptation in the harvester, (ii) development of a sampler system, and (iii) the type of sampling required for analysis. The first two requirements are fundamentally dependent on the last one.

Nawi et al. (2014) indicated that the ideal place for implementing an on-board sugarcane quality monitor would be in the elevator of the harvester, where the sugarcane material is partially cleaned and processed in the form of billets. In this context, some studies have reported promising results on the prediction of sugarcane Brix from sensor readings made on the outer-surface (‘skin’) [20] or on cross-sections [21] of sugarcane billets. Furthermore, more recent studies have advanced with on-board vis-NIR spectroscopy sensor applications on the elevator of a sugarcane harvester simulator, i.e., analyzing samples at a distance and in motion [2,22]. However, Maraphum et al. [23] and Phuphaphud et al. [24] reported that the waxy material should be removed from the cane surface for maximum accuracy in the spectral data condition, even though this may be impractical for an embedded system. Associated with this fact, Phetpan et al. [2] reported on the need to evaluate the potential of the vis-NIR spectroscopy technique with data sets consisting of a larger number of sugarcane varieties. In addition, despite the advantages of nondestructive measurement, there is a lack of basic studies comparatively evaluating various forms of sampling without and with minimal processing, using extensive numbers of samples obtained over the course of a harvest. Thus, the objective of this study was to compare different sugarcane sample types, including billets, defibrated cane, and raw juice, and to analyze the spectral response of each sampling type for the prediction of quality parameters of sugarcane from vis-NIR spectroscopy.

## 2. Materials and Methods

### 2.1. Sampling

Variability of sugarcane quality parameters throughout a harvest occurs due to diverse environmental conditions, mainly temperature and precipitation, during the harvest [25]. Based on this, the data collection procedure occurred on random periods over six months of the 2019 harvest (June to November). We sought to obtain higher variability of the sugarcane quality parameters from this collection procedure throughout the harvest.

The data collection was carried out in the quality laboratory of a sugar mill. Three hundred and two samples were collected, and different levels of processing were applied. Also, all data acquisition was performed in a controlled temperature environment (20 ± 5 °C), minimizing the effects of sugar degradation by microorganisms. In addition, we collected the daily results of conventional analysis performed by sugar mill quality laboratory over the same months in which the samples were collected for spectral analysis.

**Figure 1:**
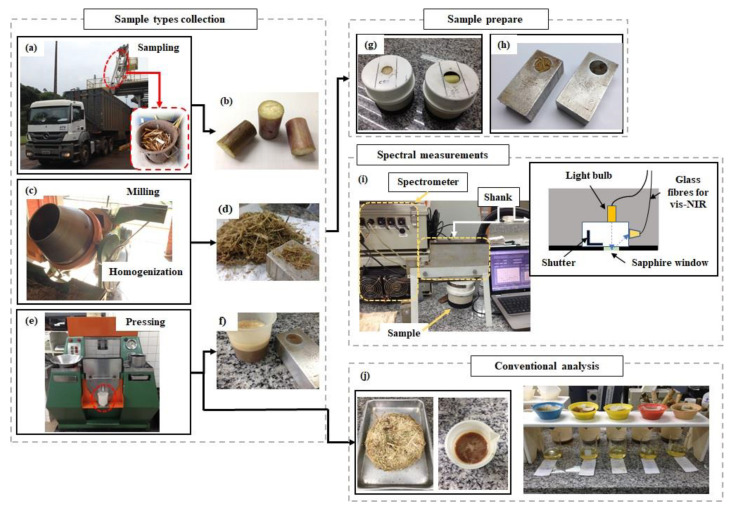
The sequence samples collection and spectral measurements during the preparation steps of samples for conventional analysis. (**a**) Sampling of sugarcane billets by an oblique probe in the cargo truck; (**b**) sugarcane billets for skin and cross-sectional scanning measurements; (**c**) milling and homogenization of sugarcane to defibrated sample; (**d**) defibration sample; (**e**) pressing of defibrated sample to extracting of juice; (**f**) extracted raw juice; prepared samples for vis-NIR spectral measurements: (**g**) cross-sectional and skin of billets inside pipeline chambers, (**h**) defibrated cane and raw juice; (**i**) Veris vis-NIR spectrometer and internal configuration scheme of the measurement shank; (**j**) fibrous cane residue and extracted raw juice for conventional analysis.

The sampling procedure for vis-NIR analysis was carried out sequentially and simultaneously to the sample preparation for conventional analysis, as described:

1. An oblique probe collected a sample of sugarcane billets in each truckload to proceed with the conventional analyzes of the sugarcane transported to the sugar mill (Figure 1a). Before the laboratory processes the sample, we took a subsample composed of three random sugarcane billets (Figure 1b);
2. The remaining sample of billets was milled in a mechanical knife crusher and homogenized in a mixer (Figure 1c). Then, samples with 500 g and 50 g of the homogenized defibrated sugarcane were collected (Figure 1d) to proceed with the conventional and spectral analysis, respectively;
3. The 500 g sample was pressed in a hydraulic press under constant pressure, at 24.5 MPa (250 kgf cm^−2^) for 60 s to obtain the raw juice for conventional analysis (Figure 1e). At the same moment, the third sample composed of extracted raw juice was collected for spectral measurements (Figure 1f).

The sample types composed of three billets, defibrated cane, and raw juice were prepared (Figure 1g,h) and immediately used for spectral measurements at the sugar mill laboratory (Figure 1i). The remains of raw juice and the bagasse without juice (after pressed) were used for conventional analytical analysis (Figure 1j).

### 2.2. Sugarcane Quality Analysis

All the procedures and equations for sugarcane quality parameters determination followed the standard protocol proposed by the CONSECANA [26]. These protocols agree with the international rules from ICUMSA.

Initially, the soluble solids content (Brix) was determined by pouring raw juice into a refractometer probe (RX-5000α, ATAGO Co Ltd., Tokyo, Japan) with a maximum resolution of 0.1 Brix. Then, 14 g of a mixture composed of a 4:2:1 proportion of Celite (mineral filtering agent), aluminum chloride, and calcium hydroxide, respectively, was added to 200 mL of raw juice homogenized by a magnetic stirrer until the solution was well-mixed. The solution was filtered through filter paper to obtaining clarified juice. A volume of 70 mL was added in a digital polarimeter (Schmidt + Haensch, Polartronic NHZ 8, Berlin, Germany) to the saccharimetric reading. The result was obtained as percentage of apparent sucrose in the juice (Pol).

After the juice extraction, the remaining fibrous cane residue (Figure 1j) was transferred to metal baskets, with holes at the base. The fibrous residue was weighed on a semianalytical balance. Then, samples were maintained in a forced air circulation dryer at a temperature of 105 °C, until constant weight was achieved. The dryer samples were weighed, and the fibre content (insoluble solids) was determined.

The Pol of cane and TRS were calculated for each sample from the previous parameters as described by CONSECANA (2015). The triplicate values obtained from each sample were averaged.

It is important to mention that reducing sugars (fructose and glucose) and purity (apparent sucrose in the soluble solids content) were also determined in the laboratory. However, while these parameters are not the subject of the present study, they were used to calculate TRS by the reference method.

### 2.3. Acquisition of Spectral Data

The spectral measurements were performed with a Veris vis-NIR spectrometer (Veris Technologies Inc., Salina, KS, USA). This equipment was developed for on-the-go soil measurements mounted on a platform, connected to a three-point hitch, and pulled by a tractor [27]. However, it may also be used in bench mode. The equipment consists of a CCD array spectrometer (USB4000, Ocean optics, Largo, FL, USA), measuring wavelengths between 373 and 1011 nm, and an InGaAs photodiode-array spectrometer (C9914GB, Hamamatsu Photonics, Hamamatsu, Japan), with a spectral range between 1170 and 2222 nm. The system presents a resolution of around 5 nm. Spectral measurement acquisitions were performed via a sapphire window in the lower of the shank using a tungsten halogen lamp as an electromagnetic energy source (Figure 1i). Each spectrum recorded by the equipment software (Veris spectrophotometer software V1.79) corresponded to the average of 20 spectral readings. The inside shutter is operated automatically to obtain dark and reference spectra before each analysis. Four external references with different grey levels were used for spectral calibration of the spectrometer before the analysis. The spectral data were stored as absorbance units.

The three billets of each sugarcane sample were cut transversely at both extremities, and their skin was lightly cleaned with paper to remove residues from harvest (Figure 1b). An ad hoc dark chamber was constructed with PVC pipes and foam to accommodate the billets, and its inside was painted matte black. Also, magnets were placed on the cover of the chamber, next to the hole through which the spectral scans were performed, aiming to fix the chamber on the reading shank of the spectrometer. This device standardizes the distance between the sapphire window of the spectrometer and the sugarcane billets and removes interference from external lighting (Figure 1g).

The spectral scan method on billets was adapted from Nawi [20,21] and Phuphaphud [15]. The spectral scans were performed at three equidistant points (around 120°) on the skin of each billet. Furthermore, the cross-sectional scanning of billets was performed in triplicate in each cross-sectional surface of each billet, only changing the position after each reading. Therefore, each sample type measurement, skin scanning (SS), and cross-sectional scanning (CSS) of billets was represented by an average of nine successive scans.

The spectral measurements of defibrated cane and raw juice were performed in the same manner. A recipient available from the equipment itself with a volume of around 3 mL was used. The recipient was filled with sample (defibrated cane or extracted raw juice, Figure 1h), and the spectral measurements were performed in triplicate. Three replicates were performed for each sample. Thus, the average of nine spectral readings of defibrated cane (DF) and nine spectral readings of raw juice (RJ) were recorded.

### 2.4. Spectral Preprocessing

Data preprocessing steps were performed to remove or minimize the sources of spectral variabilities, such as noise present in the dataset, which was not related to the analytical signal [8,28].

Firstly, the spectra were preprocessed using standard normal variate (SNV) [29] to eliminate the deviations caused by particle size and scattering, which centers each spectrum on its mean and then scales it by its standard deviation. Also, the second derivative based on the Savitzky-Golay algorithm [30] was applied, with a window size of 11 points and second-order polynomial fitting to minimize hurdles such as baseline shifts drifts and to remove high-frequency noise from a spectrum and improve the signal-to-noise ratio [28]. After the preprocessing of the spectral data, Pareto scaling (PS) was applied to variables, which is the most commonly applied scaling method in infrared data [31]. The method centered all variables at their means, and then divided them by the square root of the standard deviation.

### 2.5. Multivariate Analysis

Firstly, the spectral data of the four sampling conditions were concatenated. Then, the data was divided into calibration (75%, 227 samples) and external validation (25%, 75 samples) data sets, based on the Kennard-Stone method [19]. This procedure allowed to obtain the same samples for calibration and external validation data set for both sample types evaluated. The spectral measurements were used to build predictive models for sugarcane quality parameters based on Partial least square regression (PLS) [20].

The models were calibrated using the venetian blinds cross-validation method with 10 splits. The optimal PLS models were determined based on the lowest number of latent variables (LV), in which the root mean square error of cross-validation (RMSECV) was not significantly higher than the minimum RMSECV [23]. The root mean square error (RMSE) was calculated as follows:

(1) $$\mathrm{RMSE}=\;\sqrt{\frac{{\sum_{i=1}^{n}\left(y_{i}-\hat{y_{i}}\right)}^{2}}{n}}$$

where *n* is the number of samples, *y_i_* is the reference measurement of sample *i*, and $\hat{y_{i}}$ is the estimated result for sample *i*.

The outliers were evaluated during the calibration step for the reference lab values and spectral data. The presence of outliers in the spectral data was evaluated by the “influence plots” based on high leverage and unmodeled residuals by Hotelling T^2^ and Q statistics, respectively [8]. Samples with high values in both cases, at 5% of significance level, were considered outliers and removed from the spectral data set. On the other hand, outliers in reference data were evaluated by the root mean square error in calibration (RMSEC) values. Samples that presented errors in prediction greater than ±3 × RMSEC were considered outliers and removed from the data set [32]. The external validation samples were considered unknown samples. In this way, the outliers were evaluated only for the spectral data set. The process was carried out at most three times in the calibration step, as recommended by ASTM E1655-7 [33].

The model accuracy was evaluated based on the RMSE for calibration, cross-validation, and prediction (RMSEC, RMSECV, RMSEP, respectively). Prediction performance was evaluated based on the determination coefficient (R^2^) for calibration and prediction (R^2^c and R^2^p, respectively), and the ratio of performance to the interquartile range (RPIQ), which is calculated by the ratio between the interquartile difference and the RMSEP. Also, a randomization test [34] with 0.05 significance level of probability was performed. The aim was to compare the accuracy of regression models using different sugarcane sample types in the validation set. The hypothesis evaluated were:

- Null hypothesis (H_0_): RMSEP_sample type 1_ = RMSEP_sample type 2_ (accuracy is similar);
- Alternative hypothesis (H_1_): RMSEP_sample type 1_ ≠ RMSEP_sample type 2_ (accuracy is not similar).

An advantage of this test is its simplicity and the fact that assumptions about normality or homoscedasticity of the data are not required (distribution-free) [35]. More details about this test, included an algorithm script, can be found in Olivieri [36].

Moreover, the variable importance for the projection (VIP) was calculated to verify the wavelengths with a more significant impact on the external validate models [27] for each sample type. The VIP was calculated as follows:

(2) $${\mathrm{VIP}}_{j}=\sqrt{p\overset{h}{\underset{k=1}{\sum }}\left[Z{\left(\frac{w_{\mathrm{jk}}}{\left|\left|w_{k}\right|\right|}\right)}^{2}\right].{\left(\overset{h}{\underset{k=1}{\sum }}Z\right)}^{-1}}$$

where VIP is the variable importance for projection (dimensionless), j is a specific wavelength (nm), *p* is the number of wavelengths (dimensionless), h is the number of latent variables (dimensionless), Z is the fraction of variance in the prediction explained by the latent variable (dimensionless), and w is the loading weight (dimensionless).

All models, routines, and data processing were performed in Matlab R2015a (The MathWorks, Natick, MA, USA) and PLS Toolbox 8.9 (R8.9.1; Eigenvector Research, Wenatchee, WA, USA).

## 3. Results and Discussion

### 3.1. Overview of Sugarcane Quality Reference Data and vis-NIR Spectral Measurements of Different Sample Types

**Figure 2:**
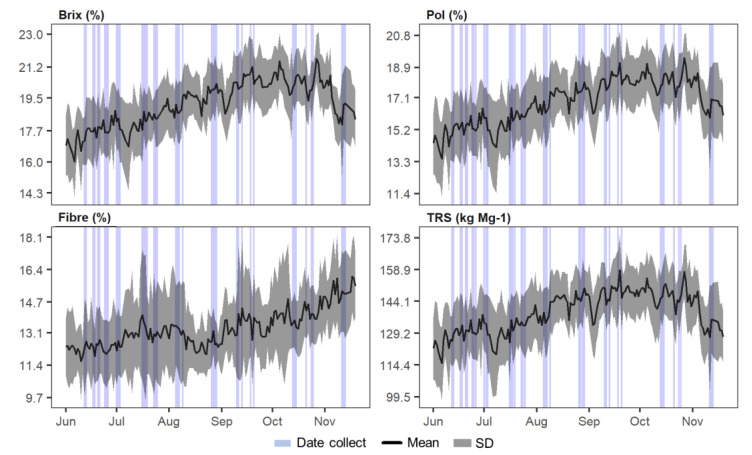
Mean and standard deviation (SD) of annual variation of sugarcane quality parameters, Brix, Pol, Fibre, and total recoverable sugars (TRS); and spectral data collection periods (vertical bars).

From the daily results of analyses performed by conventional methods at the mill, it was possible to characterize the variation of the main parameters determined analytically (Brix, Pol, and Fibre), as well as for TRS, throughout the months in which the experiment was performed (Figure 2).

It is possible to observe an increasing trend in all parameters from June to October. Afterward, there is a tendency to decrease, except for fibre. Weather is highly influential on sucrose storage [25]. In the months corresponding to autumn (June) and winter (June to September), water stress and cooler temperatures contribute to the reduction of vegetative crop growth and favor sucrose storage [37]. With the beginning of spring (September/October) and the beginning of the rainy season, the vegetative growth of the crop is resumed, and the reserves are consumed. The sample acquisition on different periods (vertical bars in Figure 2) throughout the harvest allowed us to obtain data including different stages in this variation. The effect of this variability was reflected in the range of all samples collected during the experiment (Table 1).

On a first view, the Kennard-Stone method provides a representative calibration data set, with external validation data set between its range (Table 1). The sample acquisition method provided a satisfactory variability of data, as expected; TRS varied from 86.94 to 173.80 kg of sugar per Mg of cane.

**Figure 3:**
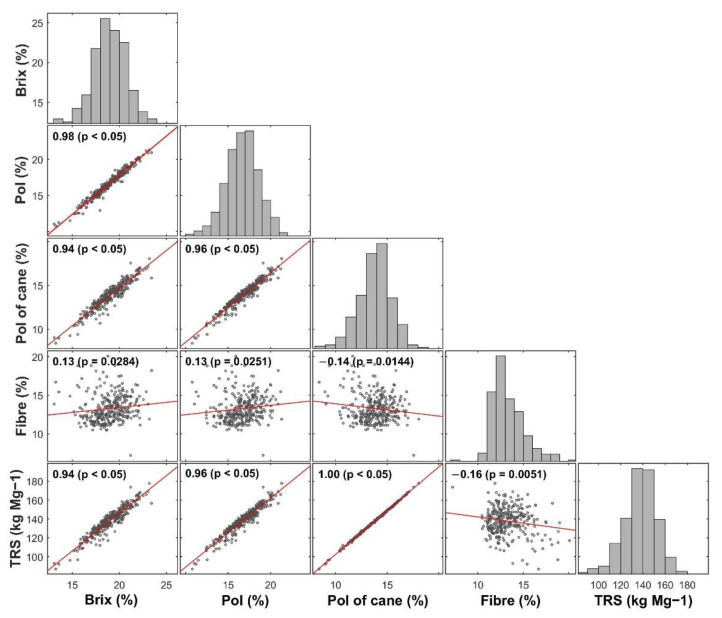
Correlogram of sugarcane quality parameters with frequency distributions on the principal diagonal, Pearson’s correlation coefficient and respective *p*-values, and the correlation trend line.

The distribution of all quality parameter values had wide distribution (Figure 3). Fibre content did not positively or negatively correlate with any other parameters analyzed, with values varying from −0.16 to 0.13 (*p* < 0.05). On the other hand, the other parameters showed a positive correlation higher than 0.94 (*p* < 0.05). The highest correlation was observed between Pol of cane and TRS, close to 1.00 (*p* < 0.05). Higher correlation values are observed between the TRS with parameters analytically determined such as Brix and Pol (0.94 and 0.96, respectively, *p* < 0.05). The correlation values for these attributes are firstly explained by the composition of the soluble solids content of sugarcane, measured by Brix, in which the largest proportion corresponds to sucrose (about 15–18%), measured by Pol [38,39], and reducing sugars (fructose and glucose) in a smaller proportion (about 0.5%) [40]; note that the determination of reducing sugars was not the objective of the present study.

**Figure 4:**
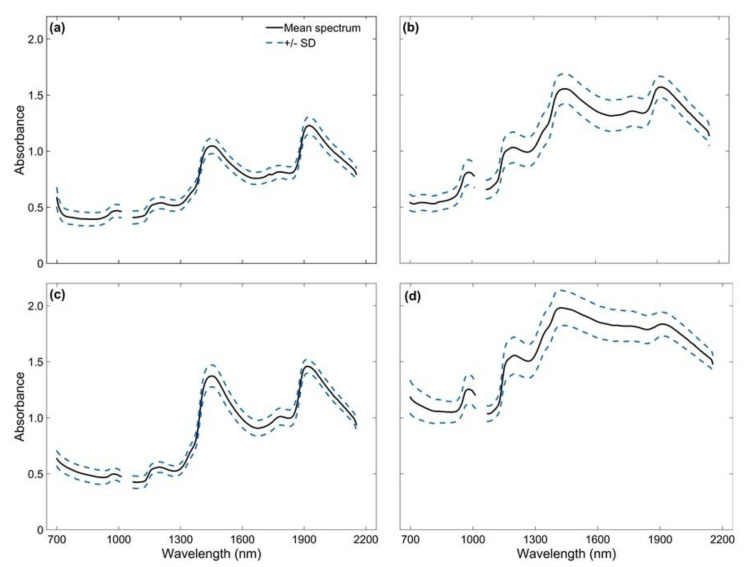
vis-NIR mean spectra and standard deviation (SD) of all 302 sugarcane samples for (**a**) skin and (**b**) cross-sectional scanning of billets, (**c**) defibrated, and (**d**) raw juice samples.

The vis-NIR raw spectral data obtained for 302 samples of each sugarcane sample type are shown in Figure 4. The spectral data were evaluated to identify possible spectral errors [41]. We observed a noisy aspect in the region corresponding to the visible spectrum (400 to 698 nm), mainly for SS samples. This effect may have been attributed to the influence of skin colors of billet samples, which were obtained for several different sugarcane varieties (Table A1), or soil residues from the harvest present in the RJ samples. Therefore, this spectral region was removed from the data set. Phuphaphud [14] observed the same effect due to the skin color of sugarcane billets. Also, based on the evaluation of the coefficient of variation (CV) obtained for each spectral band, the last spectral bands showed high CV concerning their neighbors and were also removed from the dataset, similar to the procedure performed by Franceschini [27] in a study on the external effects on the spectral reading of vis-NIR of soil samples using the same equipment. Thus, only bands in the spectral range between 699 and 1010 nm and between 1070 and 2153 nm (303 spectral bands) were retained.

**Figure 5:**
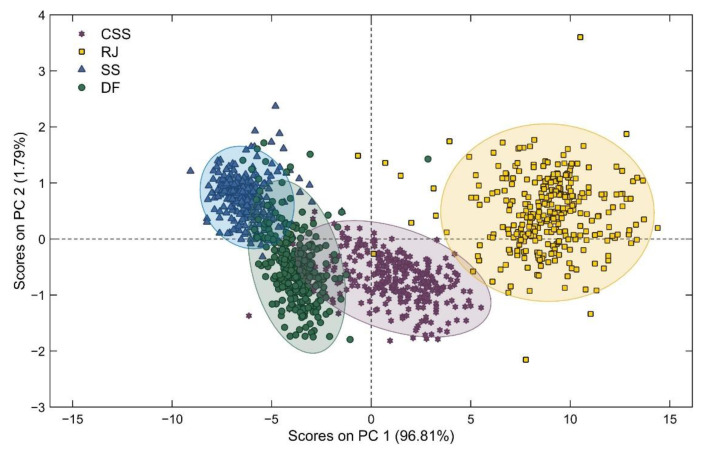
PCA score plot for the sugarcane sample types analyzed. SS-skin scanning of billets; CSS-cross-sectional scanning of billets; DF-defibrated samples; RJ-raw juice samples.

A PCA analysis performed an exploratory overview of the data structure. The spectral data were only mean-centered, and the classes were identified by sample type. Two principal components, PC1, and PC2, explained 98.6% of the data variance (Figure 5). The first component explained 96.8% of the data variance. The data structure was different for each sample type, as can be seen from ellipses illustrating the majority of samples (Figure 5). However, a first overview allowed us to verify the greater difficulty in explaining the variance of less processed samples, such as samples obtained by spectral readings in the skin (SS) and cross-sectional (CSS) of the billets, than processed samples, such as raw juice samples (RJ).

**Figure A1:**
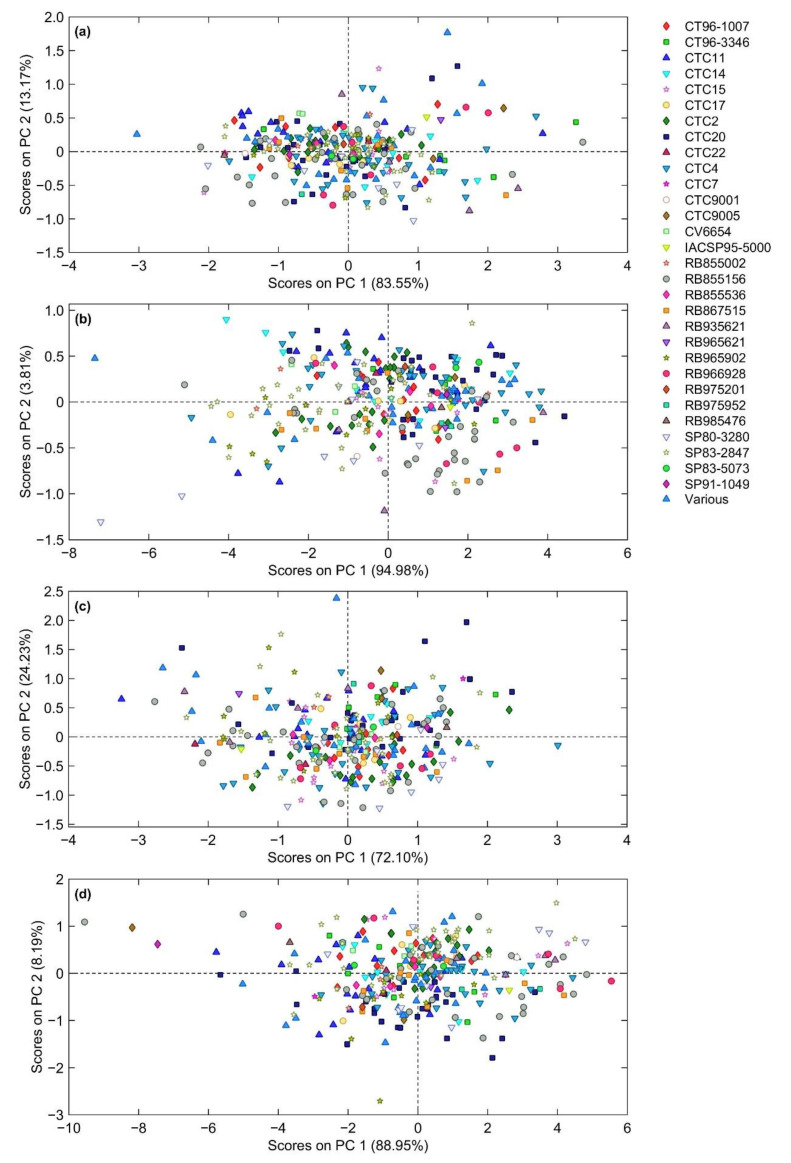
Principal component scores of different sugarcane varieties for spectral data of skin scanning of sugarcane billets (**a**), cross-sectional scanning of sugarcane billets (**b**), defibrated cane samples (**c**), and raw juice samples (**d**).

Vis-NIR spectroscopy may be used in a number of applications, including the classification of sugarcane varieties, with promising results [42]. The same authors showed that the spectral regions between 650 and 750 nm, corresponding to the visible spectrum, was the most suitable for sugarcane discrimination. The principal component analysis for the four sample types individually showed that the scatter plots were not categorized into groups based on sugarcane varieties (Figure A1). These results indicated that the sample set composed for many different varieties did not affect the spectral characteristics between each variety for both sample types. A similar effect was observed by Phuphaphud et al. (2020) [14] when evaluating the classification of three varieties. Therefore, the present study was conducted for all varieties combined.

The vis-NIR technique principle is based on the detection of compounds and molecules through their molecular vibration states [8]. Different varieties naturally have different concentrations of parameters such as sucrose and fibre according to genetics. Furthermore, for all of them, the plant matrix is essentially composed of water (75–82%), insoluble solids content (Fibre, 10–18%), and soluble solids (Brix, 18–25%), which are composed of nonsugars (1–2%), sucrose (14–24%), and reducing sugars (0–1.5%) [43]. However, the prediction of quality parameters related to chemical compounds of interest should be independent of sugarcane varieties.

### 3.2. Prediction Performance of Models Based on Different Sugarcane Sample Types

**Figure 6:**
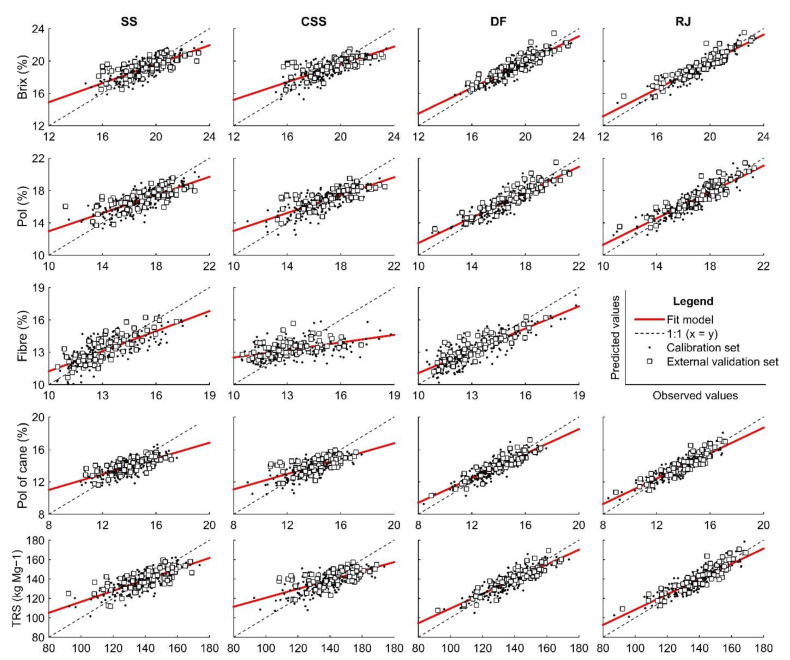
Plots of observed values versus predicted sugarcane quality values from vis-NIR by skin (SS) and cross-sectional (CSS) scanning of billets, defibrated (DF), and raw juice (RJ) samples. Brix, Pol, Fibre, and Pol of cane are in percentage, and TRS values are in kg Mg^−1^.

**Figure A2:**
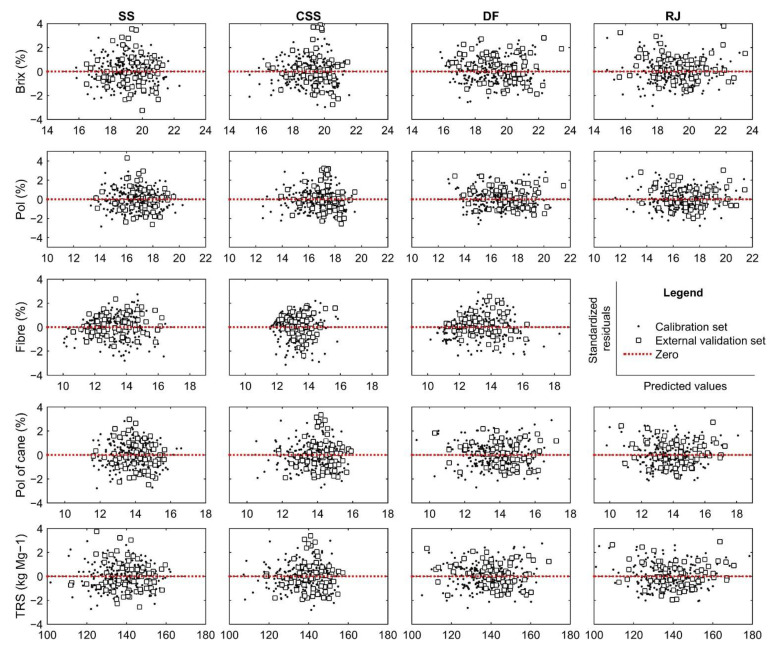
The plot of standardized residuals versus predicted sugarcane quality values from vis-NIR by skin (SS) and cross-sectional (CSS) scanning of billets, defibrated (DF), and raw juice (RJ) samples. Black dots are calibration samples, and red dots are external validation samples.

Figure 6 presents scatter plots showing reference versus predicted values of sugarcane quality parameters. There was an underestimation of high values and overestimation of lower values for all attributes and sample types evaluated. However, this effect was more intense for the less prepared sampling condition, such as SS and CSS. Also, overall, the residuals showed no trend (Figure A2).

More LVs were necessary to explain the variance of the data for models constructed from SS samples (between 7 and 10) than those obtained to predict the same parameters from other sample types (Table 2). Also, it could be observed that SS and CSS did not show similar accuracy (*p*-value < 0.05) for Fibre, Pol of cane, and TRS (Table 3). Moreover, the prediction performance results for these parameters by these sample types were worse than the performance results obtained by DF and RJ samples (Table 3). The RPIQ values for TRS were 40% higher on average than those for SS and CSS samples, for example. Furthermore, the model accuracy observed between sampling methods for all the other conditions was statistically nonsignificant (*p*-value > 0.05).

The model performance for DF and RJ samples was equivalent for practically all parameters evaluated. There was no significant difference between the model’s accuracy (*p*-value > 0.05) and very close values of R^2^p and RPIQ. Moreover, from DF samples, it was possible to obtain a satisfactory performance to predict Fibre content; this was not possible for RJ samples. On the other hand, the models performed for SS samples presented a higher number of LV than for a prepared sample. The model performance for predicting parameters related to sucrose (Brix, Pol, Pol of cane, and TRS) was not satisfactory, with R^2^p and RPIQ below 0.5 and 2.0, respectively, except for Fibre prediction. The prediction results from SS samples for Fibre were close to those obtained for DF samples, as shown by the values of R^2^p and RPIQ. However, the results were less promising than those obtained by Phuphaphud et al. (2019) [15], which obtained the following results: maxima of 0.81 for R^2^p and 0.63 for RMSEP. Although Fibre content is an important attribute for sugarcane quality determination, it is not essential for sucrose estimation. Fibre content has no relation with some important attributes, such as Brix and Pol, and only minimally impacts TRS calculation. The prediction of this parameter is important for producing energy cane and breeding programs, as in work developed by Phuphaphud et al. (2019) [14].

Some models developed for CSS samples were similar to those developed for SS, as for Brix and Pol prediction. However, its predictive performance was lower than those obtained for Fibre, Pol of cane, and TRS predicted by SS samples, with worse results for R^2^p and RPIQ. In a first investigation, Nawi et al. (2013a) obtained values of 0.87 for R^2^p. The excellent performance of this index can be explained by the method of data acquisition adopted by the authors, with individualized samples according to the stem portion (lower, middle, and upper portion) and only three varieties of cane. Sucrose accumulation occurs in an ascending manner, with more accumulation in the lower portion and less in the internodes of the upper portion, close to the leaves [38,44,45]. Therefore, samples composed of different sections resulted in more variability in quality parameters. However, if we analyze the characteristics of a sugar cane harvester, after the stems pass through the chopper roll system, the distinction between portions of the sugarcane stem is not viable.

The RPIQ values for the SS method were higher than those obtained by the CSS method. The SS method on billet samples on the harvester conveyor would be the most practical method, due to the better operability of sample acquisition in that portion of the harvester [16]. However, several external factors must be considered to measure quality attributes by the SS method. A critical one is the constitution of the sugarcane skin itself, as various colors depending on the variety, black and white waxy material, and organic compounds may be present [24,46].

There are common waxy materials on the cane surface that affect vis-NIR measurements by the SS method. Maraphum et al. (2018) evaluated the effect of the waxy material on the cane surface to eliminate or avoid getting low accuracy of the models for Pol measurements. They obtained RMSEP values were around 1.20 to 1.50%, i.e., close to those found by the present study. The authors concluded that spectra acquisition by removed-wax samples was convenient for the measurement of Pol. However, other compounds could affect vis-NIR spectroscopy measurements, such as cellulose and lignin [7].

Overall, the predictive performance results of the models (based on the R^2^p and RPIQ) indicate that DF and RJ samples presented similar performance and provide the best results. However, models built from DF samples require less preparation, i.e., by avoiding juice extraction, making them more attractive for an on-board system. Moreover, CSS samples presented worse performance than all other samples. SS samples presented higher values of R^2^p and RPIQ than CSS samples for all quality parameters. Furthermore, SS samples showed a nonsignificant difference (*p*-value > 0.05) of accuracy (RMSEP) with models built from DF samples, but worse results for performance (R^2^p and RPIQ), except for Fibre. Possibly DF results were satisfactory due to the exposure of the internal constituents, which overlapped concerning the waxy material that becomes visually negligible. On the other hand, the organic compounds in the sugarcane outer-surface may have interfered in the prediction models. Future studies using nonlinear processing methods [47] or advanced filtering methods, with orthogonalization of unwanted signals concerning the compounds of interest [48], may help in increasing the predictive performance of the models, which is more interesting for an on-board system.

### 3.3. Variable Influence on the Models

**Figure 7:**
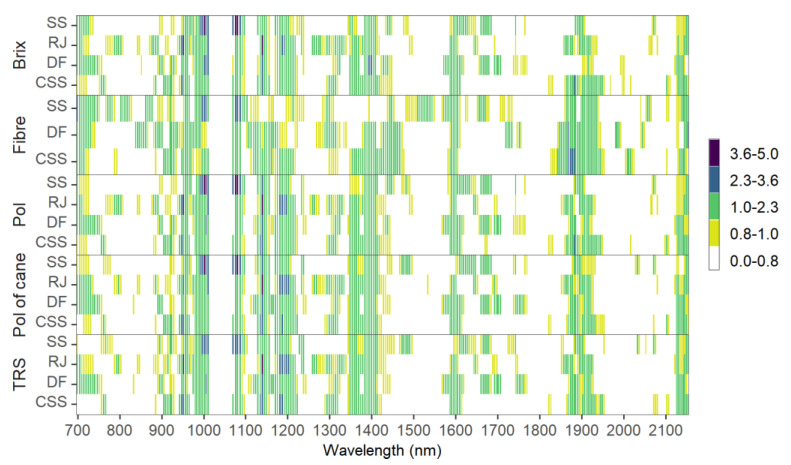
Heatmap of Variable Importance in Projection (VIP) for models used to predict Brix, Pol, Fibre, Pol of cane, and TRS based on different spectral sample types datasets. SS: skin scanning of billets; CSS: cross-sectional scanning of billets; DF: defibrated samples; RJ: raw juice samples; TRS–total recoverable sugars.

VIP scores were used to describe the importance of each wavelength to the prediction of the main sugarcane quality parameters (Figure 7).

As a vibrational spectroscopy technique, the interaction between the vis-NIR electromagnetic radiation and the matter of the sample could be interpreted mainly by overtones and combinations of vibrational modes involving C-H, O-H, and N-H chemical bonds [8,49]. VIP values greater than 1.0 indicate variables with greater influence on the models, and VIP values between 0.8 and 1.0 indicate the moderately influential variables. All variables with VIP smaller than 0.8 are insignificant to the predictions [50]. Some substantial similarities could be observed for different samples and quality parameters on a first overview.

At around 960 nm, there is a small interval with high values of VIP (higher than 2.3), especially related to the prediction of Brix, Pol, Pol of cane, and TRS by CSS and RJ samples, corresponding to the second and third overtone of O-H and C-H stretching, respectively [51]. The region between 980 and 1030 nm can be regarded as an important contributor to quality predictions (VIP higher than 1.0). Between 972 and 1009 nm there is a characteristic signal related to saccharides and the third overtone of O-H [7]. This signal is more expressive for parameters determined for SS samples. This spectral range could be associated with cellulosic fibres, which explained the higher VIP values to SS samples. Similar observations were found by Phuphaphud et al. (2020) to predict commercial cane sugar from growing cane stalks for breeding programs using vis-NIR spectroscopy.

At 1139 nm, there is a small band with VIP values higher than 1.0 for all parameters predicted for four sample types, except for Fibre content. On the interval between 1100 and 1230 nm occurs the second vibrational frequency overtones associated with C-H stretching [51]. Also, at around 1170–1180 nm, there are VIP values higher than 1 for Fibre predicting, mainly by DF and CSS samples. In this region, the third overtone of C-H and unsaturated C=C double bonds are typically associated with fibre, such as lignin [7].

At 1360 nm, there is another expressive region with high VIP values, possibly related to C-H combinations and the O-H first overtone, respectively [49]. Then, at 1600 nm, there are highly similar VIP values possibly associated with to first vibrational frequency overtone of C-H stretching [51]. Another region shows a high contribution, with VIP values higher than 1, for Fibre predicting between 1850 and 1900 nm, mainly for CSS and DF samples. Around 1820 nm occurs the effects of O-H stretching associated with two combinations of C-O stretching commonly associated with Fibre as cellulosic [7]. This interval is lower and with lower VIP values for SS samples, possibly due to the waxy effect on the near-infrared signal [23]. Finally, in the last bands of the spectra, after 2100 nm, the intensity of VIP values is similar for all predicted attributes from any sample type due to O-H bending and C-O stretching combination [7].

**Figure A3:**
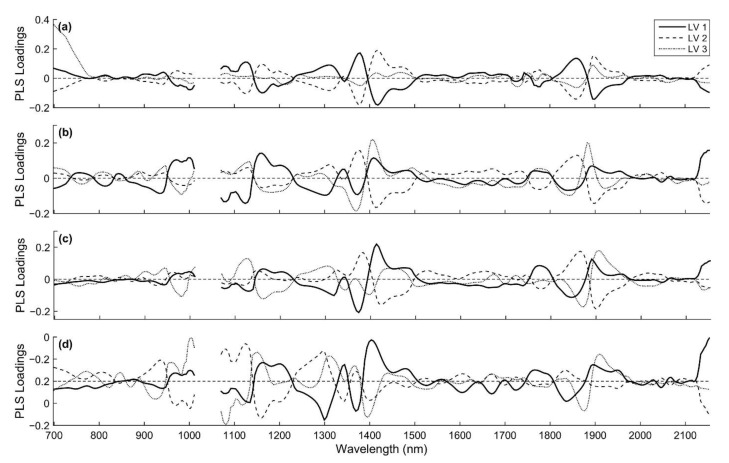
The three first Partial least squares loadings for Brix prediction using near-infrared reflectance spectroscopy from the skin (**a**) and cross-sectional (**b**) scanning of billets, defibrated cane (**c**), and extracted raw juice (**d**).

**Figure A4:**
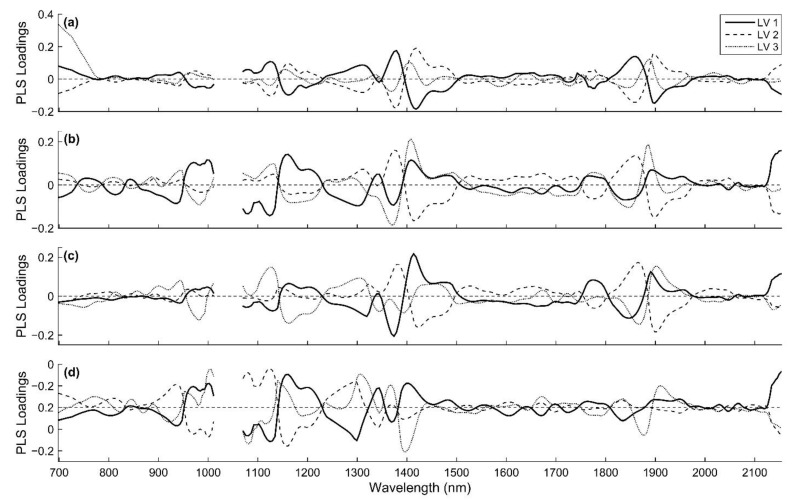
The three first Partial least squares loadings for Pol prediction using near-infrared reflectance spectroscopy from the skin (**a**) and cross-sectional (**b**) scanning of billets, defibrated cane (**c**), and extracted raw juice (**d**).

**Figure A5:**
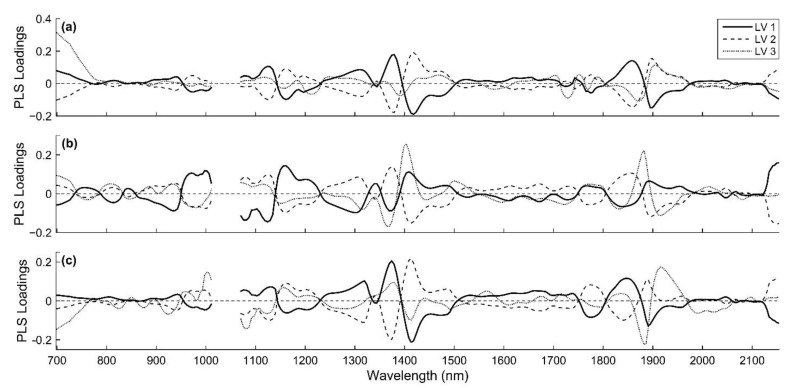
The three first Partial least squares loadings for Fibre prediction using near-infrared reflectance spectroscopy from the skin (**a**) and cross-sectional (**b**) scanning of billets, and defibrated cane (**c**).

**Figure A6:**
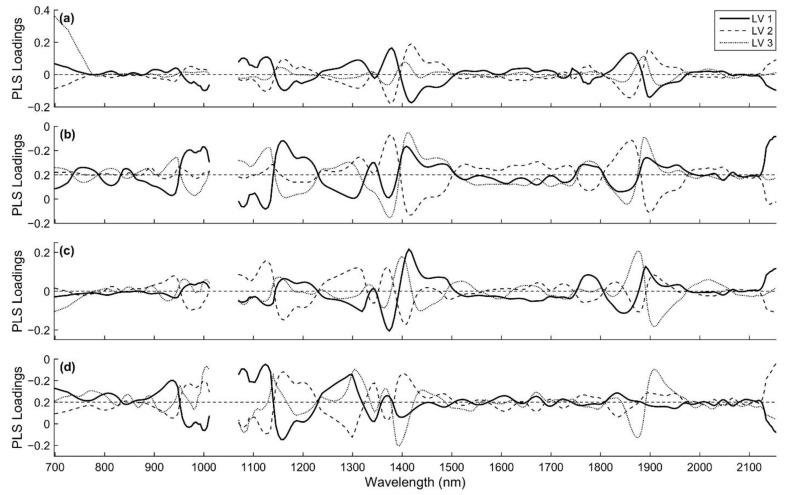
The three first Partial least squares loadings for Pol of cane prediction using near-infrared reflectance spectroscopy from the skin (**a**) and cross-sectional (**b**) scanning of billets, defibrated cane (**c**), and extracted raw juice (**d**).

**Figure A7:**
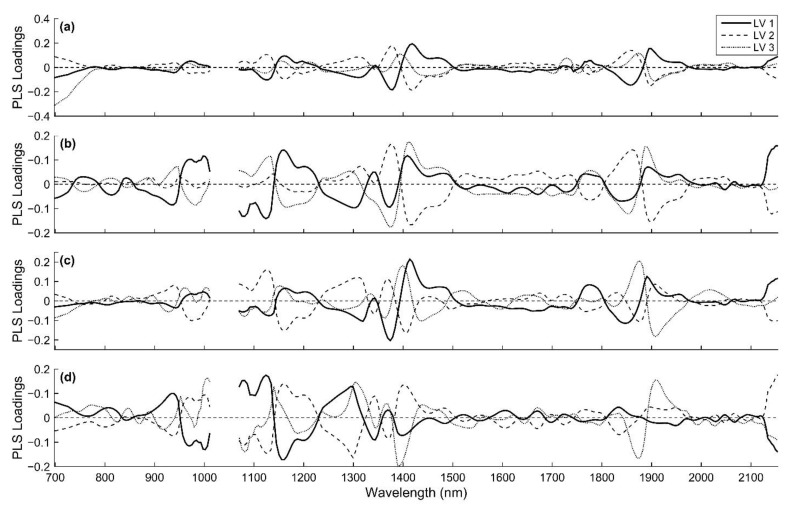
The three first Partial least squares loadings for total recoverable sugars (TRS) prediction using near-infrared reflectance spectroscopy from the skin (**a**) and cross-sectional (**b**) scanning of billets, defibrated cane (**c**), and extracted raw juice (**d**).

The scores of the models for all measured quality parameters are displayed by their first PLS loadings (Figure A3, Figure A4, Figure A5, Figure A6 and Figure A7 see Appendix A), accounting for more than 95% of the data variance. Overall, the most considerable variation occurred in the spectral region between 1300 and 1500 nm and between 1800 and 1950 nm. Other authors have found similar response in these spectral regions for prediction of sugars in other products [52,53]. This effect was similar for all sample types and all parameters evaluated. Therefore, this fact proved the relationships identified by the VIP scores and the key molecular bonds related to the parameters of interest described earlier.

The gap between two spectrometers, starting at 1011 nm until 1070 nm, is not related to significative known vibrational frequency overtones associated with some bands related to sugars or fibres [7]. Therefore, the absence of information in this range would not have significantly affected the development of the models.

Processed samples allowed a more significant interaction of electromagnetic radiation corresponding to vis-NIR bands with matter constituents. This physical effect resulted in more prominent signals from specific vibrational frequency bands related to the chemical constitution of sugarcane quality parameters. Overall, defibrated samples (DF) showed performance prediction results that were close to raw juice samples (RJ). Also, the DF sample allowed us to predict Fibre content as well as other parameters, which is not possible with RJ samples. The prediction of sugarcane quality parameters from less processed samples is a desirable characteristic for mechanization of on-the-go measurements of crops, thus promoting spatial information of crops based on quality. DF samples may partially satisfy this requirement; however, this is a destructive sampling technique.

Improving the predicting performance of sugarcane quality parameters from billets for on-the-go systems may be possible [20]. Some effects, such as waxy and skin organic compounds, need to be considered and minimized [24]. Other data processing techniques such as nonlinear models [47] or advanced filtering methods such as orthogonalization [48], could be investigated to improve the performance aiming to develop reliable models for measuring sugarcane quality using billets of cane.

## 4. Conclusions

This study demonstrates that vis-NIR spectroscopy could be used as a quick method to assess the abundance of chemical compounds of sugarcane related to its quality. There was no significant difference (*p*-value > 0.05) in the accuracy (RMSEP) of prediction of whole cane samples when compared to processed samples, such as defibrated cane (DF) and extracted raw juice (RJ), for all evaluated quality parameters. Also, outer-surface measurements of sugarcane billets presented a better accuracy (RMSEP, *p*-value > 0.05) and performance (R^2^p and RPIQ) than measurements on the cross-section.

Despite the similar accuracy (*p*-value > 0.05), DF and RJ sampling presented better performance than outer-surface measurements of sugarcane billets. Moreover, the performance of the models from DF and RJ samples were similar, but DF samples involve less preparation, as they do not require juice extraction of the sample.

The results showed that DF sampling could be used to predict the main sugarcane quality parameters, such as soluble solids content (Brix), saccharose (Pol), Fibre, Pol of cane, and total recoverable sugars (TRS), all of which are used for pricing and trading between mills and sugarcane producers. The DF models presented RMSEP varying between 0.72% and 0.87% for Brix, Pol, Fibre, and Pol of cane, and 6.71 kg Mg^−1^ for TRS.

The results in this study contribute to advancing the development of on-board quality monitoring in sugarcane. This information shows the spatial variability of crop quality and helps guide site-specific management of sugarcane fields.

## Figures and Tables

**Table 1:** Descriptive statistics of the reference results for the sugarcane quality attributes of all samples, calibration, and external validation data sets.

Parameter	unit	Mean ± SD	Median	Range	p_25_	p_75_	SEL
All samples (*n* = 302)							
Brix	%	18.95 ± 1.71	18.99	13.08–23.42	17.80	20.01	0.03
Pol	%	16.67 ± 1.90	16.66	10.78–21.20	15.41	17.95	0.01
Fibre	%	13.29 ± 1.79	12.90	7.22–20.08	12.07	14.33	0.07
Pol of cane	%	13.80 ± 1.56	13.91	8.40–17.56	12.92	14.78	0.01
TRS	kg Mg^−1^	137.66 ± 14.48	138.66	86.94–173.80	129.75	146.84	1.12
Calibration set (*n* = 227)							
Brix	%	18.86 ± 1.66	18.80	13.08–23.42	17.79	19.98	-
Pol	%	16.54 ± 1.86	16.55	10.78–21.20	15.38	17.78	-
Fibre	%	13.31 ± 1.89	12.83	7.22–20.08	12.05	14.41	-
Pol of cane	%	13.69 ± 1.52	13.79	8.40–17.56	12.83	14.60	-
TRS	kg Mg^−1^	136.65 ± 14.07	137.01	86.94–173.80	128.99	145.19	-
Validation set (*n* = 75)							
Brix	%	19.24 ± 1.85	19.59	13.55–23.05	18.06	20.61	-
Pol	%	17.06 ± 1.98	17.33	11.24–20.90	15.73	18.54	-
Fibre	%	13.23 ± 1.44	13.02	10.49–17.15	12.24	14.16	-
Pol of cane	%	14.14 ± 1.64	14.34	8.96–17.14	13.27	15.47	-
TRS	kg Mg^−1^	140.76 ± 15.36	142.59	92.16–169.02	132.35	152.44	-

SD: standard deviation; p_25_: lower quartile; p_75_: upper quartile; SEL: standard error of laboratory; TRS: total recoverable sugar.

**Table 2:** Figures of merit for the PLSR models for all studied sugarcane quality attributes and sample types.

Attribute	Sample Type	LV	RMSEC ^a^	RMSECV ^a^	RMSEP ^a^	R^2^c	R^2^p	RPIQ
Brix	SS	9	0.92	1.10	1.29	0.64	0.48	1.98
	CSS	6	0.95	1.04	1.38	0.62	0.41	1.85
	DF	7	0.67	0.75	0.84	0.81	0.80	3.05
	RJ	8	0.64	0.83	0.75	0.85	0.85	3.39
Pol	SS	8	1.09	1.26	1.42	0.60	0.48	1.98
	CSS	6	1.09	1.19	1.44	0.61	0.44	1.95
	DF	7	0.82	0.93	0.87	0.79	0.83	3.24
	RJ	7	0.80	0.97	0.90	0.82	0.81	3.12
Fibre	SS	10	1.02	1.29	0.87	0.59	0.65	2.22
	CSS	4	1.45	1.50	1.27	0.24	0.23	1.51
	DF	5	0.93	1.04	0.82	0.69	0.69	2.36
	RJ ^b^	-	-	-	-	-	-	-
Pol of cane	SS	7	0.95	1.07	1.13	0.52	0.46	1.94
	CSS	5	1.01	1.09	1.27	0.52	0.31	1.73
	DF	7	0.73	0.84	0.72	0.76	0.81	3.04
	RJ	7	0.71	0.85	0.72	0.78	0.81	3.07
TRS	SS	9	8.57	10.27	10.86	0.60	0.50	1.85
	CSS	5	9.49	10.17	11.86	0.50	0.32	1.69
	DF	7	6.50	7.51	6.71	0.76	0.82	2.99
	RJ	7	6.38	7.95	6.79	0.78	0.81	2.96

SS: skin scanning of billets samples; CSS: cross-sectional scanning of billets samples; DF: defibrated samples; RJ: raw juice samples. LV: latent variable. RMSEC: root mean square error of calibration. RMSECV: Root Mean Square Error of Cross-Validation. RMSEP: Root Mean Square Error of Prediction. R^2^c: calibration coefficient of determination. R^2^p: prediction coefficient of determination. RPIQ: Ratio of performance to interquartile distance. ^a^ values for Brix, Pol, Fibre, and Pol of cane are in percentage and TRS in kg Mg^−1^. ^b^ The fibre content was not determined from raw juice samples.

**Table 3:** *p*-Values of randomization test of external validation set for all compared sugarcane sample types.

Binary Combination (Sample Types)	Sugarcane Quality Parameters				
	Brix	Pol	Fibre	Pol of cane	TRS
SS vs. CSS	0.104	0.116	<0.001	0.036	0.008
SS vs. DF	1.00	1.00	0.667	1.00	1.00
SS vs. RJ	1.00	1.00	-	1.00	1.00
CSS vs. DF	1.00	1.00	1.00	1.00	1.00
CSS vs. RJ	1.00	1.00	-	1.00	1.00
DF vs. RJ	0.879	0.344	-	0.606	0.502

SS: skin scanning of billets; CSS: cross-sectional scanning of billets; DF: defibrated samples; RJ: raw juice samples; TRS: total recoverable sugar.

## Data Availability

The data presented in this study are available on request from the corresponding author.

## Appendix A

**Table A1:** Number of samples of each Brazilian sugarcane variety used in the study.

Variety	# Samples	Variety	# Samples	Variety	# Samples
CT96-1007	12	CTC 9001	1	RB966928	7
CT96-3346	7	CTC 9005	2	RB975201	1
CTC 11	19	CV 6654	3	RB975952	2
CTC 14	10	IACSP95-5000	1	RB985476	1
CTC 15	12	RB855002	3	SP80-3280	9
CTC 17	7	RB855156	33	SP83-2847	31
CTC 2	19	RB855536	4	SP83-5073	2
CTC 20	26	RB867515	9	RB965621	1
CTC 22	2	RB935621	4	SP91-1049	1
CTC 4	34	RB965621	1	Various	26
CTC 7	1	RB965902	12		
